# Implantable NMR Microcoils in Rats: A New Tool for Exploring Tumor Metabolism at Sub-Microliter Scale?

**DOI:** 10.3390/metabo11030176

**Published:** 2021-03-17

**Authors:** Justine Deborne, Noël Pinaud, Yannick Crémillieux

**Affiliations:** Institut des Sciences Moléculaires, Université de Bordeaux, 33076 Bordeaux, France; justine.deborne@u-bordeaux.fr (J.D.); noel.pinaud@u-bordeaux.fr (N.P.)

**Keywords:** magnetic resonance spectroscopy (MRS), metabolic imaging, implantable microcoil, brain spectroscopy, tumor metabolism

## Abstract

The aim of this study was to evaluate the potential of a miniaturized implantable nuclear magnetic resonance (NMR) coil to acquire in vivo proton NMR spectra in sub-microliter regions of interest and to obtain metabolic information using magnetic resonance spectroscopy (MRS) in these small volumes. For this purpose, the NMR microcoils were implanted in the right cortex of healthy rats and in C6 glioma-bearing rats. The dimensions of the microcoil were 450 micrometers wide and 3 mm long. The MRS acquisitions were performed at 7 Tesla using volume coil for RF excitation and microcoil for signal reception. The detection volume of the microcoil was measured equal to 450 nL. A gain in sensitivity equal to 76 was found in favor of implanted microcoil as compared to external surface coil. Nine resonances from metabolites were assigned in the spectra acquired in healthy rats (n = 5) and in glioma-bearing rat (n = 1). The differences in relative amplitude of choline, lactate and creatine resonances observed in glioma-bearing animal were in agreement with published findings on this tumor model. In conclusion, the designed implantable microcoil is suitable for in vivo MRS and can be used for probing the metabolism in localized and very small regions of interest in a tumor.

## 1. Introduction

Magnetic resonance spectroscopy (MRS) is a complementary technique to magnetic resonance imaging (MRI) for the study of brain pathologies and, in particular, brain tumors. Tumor classification, grade determination and tumor metabolism remain essential questions for prognosis and therapeutic management (radiotherapy, neurosurgical resection, etc.).

As a matter of fact, ${}^{1}$H MRS is a valuable method to assess the main brain metabolic biomarkers: N-acetylaspartate (NAA), total choline (tCho) and total creatine (tCr) compounds, lactate (Lac), and mobile lipids; and the observation [1] that brain tumors present a very different spectrum from the healthy brain [2] has allowed the positioning of ${}^{1}$H MRS as a solid contribution to tumor diagnosis. Möller-Hartman et al. [3] showed that ${}^{1}$H MRS increased diagnostic relevance and efficiency of a brain tumor by 16% compared to morphological MRI alone and Doblas study [4] demonstrates the potential of ${}^{1}$H MRS for the characterization and the differentiation of several rodent glioma models. However, nuclear magnetic resonance (NMR) spectroscopy remains a technique with marked limitations for the study of small volumes or finite quantities of materials, as may be the case for some primary tumors and metastases.

The use of miniaturized coils represents one of the most efficient solutions, with the increase of the static magnetic field, to perform spectroscopy or magnetic resonance imaging on these samples [5]. Indeed, as a first approximation, the sensitivity of an NMR coil scales linearly with the inverse of the coil size [6]. In addition, reducing the detection volume of the coil allows the filling factor of the coil to be optimized. Very few studies using implantable microcoils with diameters below several millimeters have been reported [7,8] and the in vivo one has focused on water signal detection due to very small volume of detection and limited level of signal-to-noise ratio (SNR) [8]. When the dimensions and geometry of the microcoils are suitable, one can envision to take advantage of their sensitivity to perform MRI or MRS measurements in situ in biological tissues. The implantation of microcoils in situ would thus make it possible to acquire spectroscopic or imaging data with a sufficient signal-to-noise ratio (SNR) on a small volume of interest representative of a tumor.

The implantation of the microcoil imposes strong geometrical constraints to limit invasiveness and preserve the healthy and pathological tissues to be investigated. It is essential, for example, for implantable devices to have a needle shape with a diameter limited to a few hundred micrometers. In addition, the implantable device must be sufficiently rigid to allow its insertion into the tissue.

Thus, it is therefore important to find a compromise for the design of the implantable microcoil to satisfy the minimally invasive character, the size of the volume of interest and the available SNR. Finally, it is necessary to limit the distortions of the static magnetic field by ensuring the best possible match between the magnetic susceptibility of the surrounding tissue and that of the coil.

In this study, we evaluated the performance of an implantable microcoil designed to obtain diagnostic-quality in vivo proton NMR spectra in healthy rats and C6 glioma-bearing rats. In order to preserve tissue during the implantation of the microprobe, we opted for an elongated coil with a diameter limited to a few hundred micrometers. The implantable microcoils were manufactured using insulated copper microwires (150 micrometer diameter) adapted to match the magnetic susceptibilities of the tissue. In this article, we detail the manufacturing process of the implantable microcoils and the explorations of the results obtained in vitro and in vivo in rats.

## 2. Results

### 2.1. In Vitro Results

**Figure 1:**
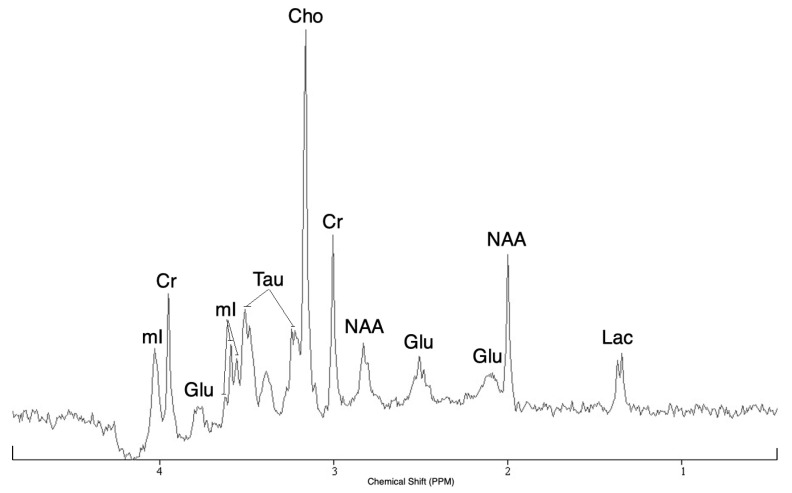
In vitro ${}^{1}$H spectrum of the solution of 7 brain metabolites with a concentration fixed at 25 mM acquired with the microcoil (detection volume: 0.450 μL) at 7 T using the PRESS sequence (TR/TE = 2000/15 ms, Nacc = 256, Tacq = 8 min 32 s).

A case of spectrum of 7 brain metabolites (Cr, Cho, Glu, Lac, mI, NAA, Tau) acquired with the microcoil with the PRESS sequence in an acquisition time of 8 min (256 averages) is shown in Figure 1.

Based on the characteristics of the different metabolites studied [9], the intensities of the resonance peaks correspond to expectation.

The good spectral quality, assessed with signal-to-noise ratios (SNR) and full widths at half maximum (FWHM), obtained allows to have not overlapping resonance peaks and to observe the J-coupling of the doublet of the lactate (line splitting of 7 Hz). The average value of the FWHM for the four singlets of the spectrum (Cr at 3.91 ppm, Cho at 3.18 ppm, Cr at 3.02 ppm and NAA at 2 ppm) is 5.1 Hz.

From the estimated SNR values, the sensitivity values S${}_{c}$, S${}_{m}$ and the limits of detection nLOD${}_{c}$ and nLOD${}_{m}$ were calculated for each metabolite of interest (lactate doublet, creatine, choline and NAA) and compared for the two types of coils, and presented in Table 1. The corresponding factor of gain (FOG) of the implantable microcoil are given as well for these NMR resonances in Table 2.

The values, presented in the tables below, show that the use of the microcoil has a strong advantage when detecting low quantities of metabolites in small volumes of interest (below microliter) despite a significantly smaller detection volume. The FOG of the implantable microcoil, ratio of the nLOD${}_{m}$ values, taking into account the respective detection volume of the two coils illustrates more particularly the gain in sensitivity per unit of volume brought by the use of the microcoil. The average FOG for the five resonance lines is equal to 76.

### 2.2. In Vivo Results

**Figure 2:**
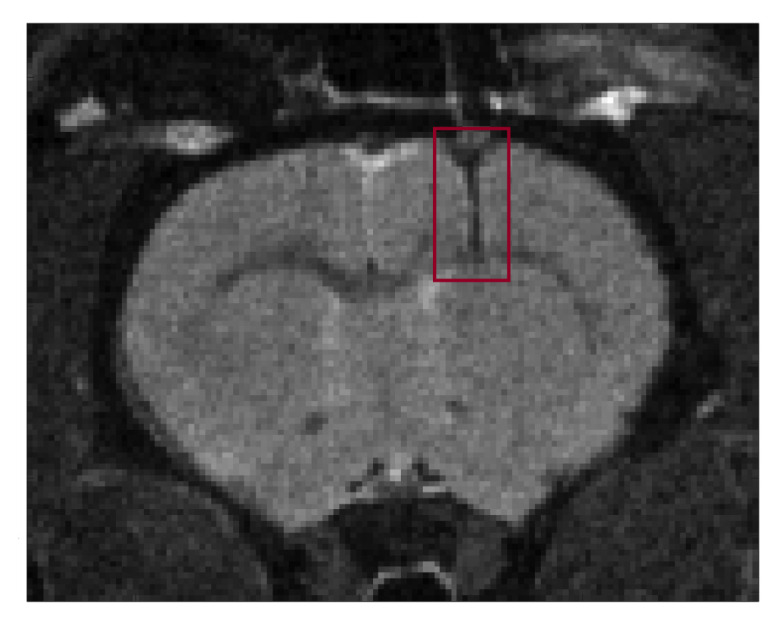
Visualization of the implantable microcoil and the PRESS voxel in anatomical T${}_{2}$-weighted MR image of the healthy rat brain.

An anatomical MR T${}_{2}$-weighted image of the healthy rat brain shows the positioning of the implantable microcoil and the acquisition voxel (Figure 2).

**Figure 3:**
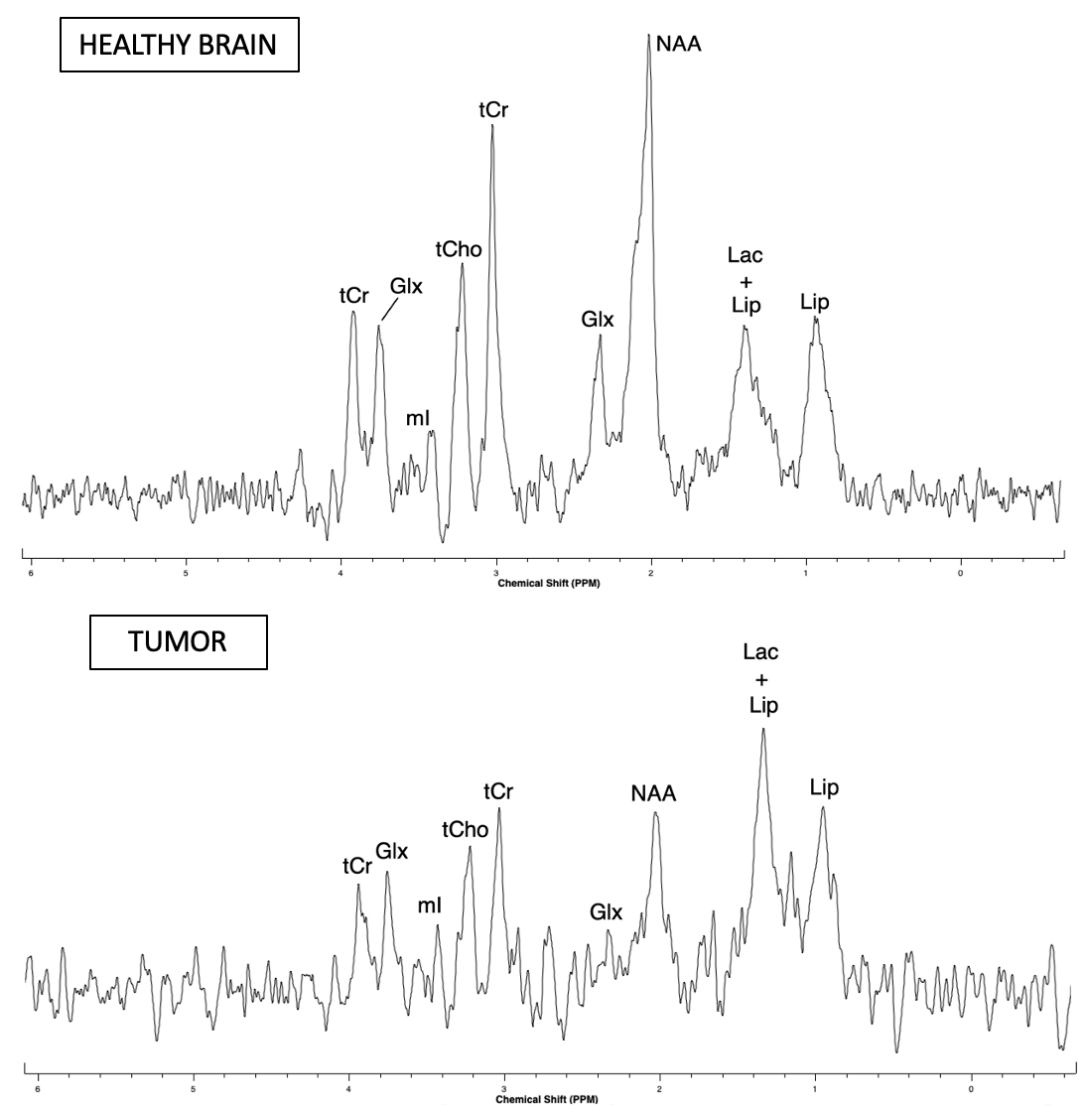
In vivo ${}^{1}$H magnetic resonance spectroscopy (MRS) spectra of healthy rat brain and tumor of the C6 tumor model acquired with the microcoil (detection volume: 0.450 μL) at 7 T using the PRESS sequence (TR/TE = 2000/15 ms, Nacc = 512, Tacq = 17 min 04 s). Assignments of resonances as indicated in Figure 3 are total choline (tCho: GPC (glycerophoshorylcholine) + PCho (phosphorylcholine) + Cho (free choline)), total creatine (tCr: PCr (phosphocreatine) + Cr (creatine)), glutamate/glutamine (Glx), lactate (Lac), N-acetylaspartate (NAA) and lipids (Lip). NB: The vertical scale of the two spectra is different.

Representative examples of postprocessed NMR spectra acquired in the right cortex of the healthy rat and in the tumor of the C6 glioma-bearing rat using an implanted microcoil is shown in Figure 3.

For the spectrum acquired in healthy rat, the FHWM and SNR of the tCr resonance were, FWHM: 18 Hz, SNR: 18 and for the spectrum acquired in the tumor-bearing rat, the FHWM and SNR of the tCr resonance were, FWHM: 21 Hz, SNR: 8. Note that a two-fold lower SNR of water peak, attributed to mis-optimized coil tuning and matching, was noticed in the tumor-bearing rat.

Qualitative comparison of spectra obtained in the healthy rat brain and in the glioma-bearing rat indicates a relative decrease of NAA peak intensity as compared to those of tCr and tCho peaks. Lactate and lipid peaks show an increase in intensity as compared to the rest of the peaks in the spectrum. These metabolic and spectral observations are in good agreement with those previously reported in the literature on the C6 glioma model [10].

A good reproducibility of the MRS acquisitions in the five healthy rats were observed with mean values and standard deviations (SD) for the tCho/tCr, tCho/NAA and NAA/tCr ratios respectively equal to 0.16 +/− 0.08, 0.15 +/− 0.06 and 1.06 +/− 0.16. The mean value and the SD of the FHWM and SNR of the tCr resonance were equal to, FWHM: 18 ± 1 Hz and SNR: 20 ± 2.

## 3. Discussion

The compromise found between the design (minimally invasive feature) and the optimized use (best quality of NMR results) of the microcoil resulted in the ability of identifying, allowed by a sufficiently good spectral resolution, and quantifying, allowed by a high enough SNR, some metabolites such as NAA, Cho, Cr and Lac in healthy tissue and tumor tissue.

The brain implantation of the microcoil did not produce any bleeding. No inflammation or hemorrhage was observed in the MRI images and spectra obtained with the implanted microcoils were similar to those obtained with conventional external coils. The quality of spectra obtained with the microcoil is actually a good sign of the limited impact of the implantation of the coil on the tissue integrity. These observations are consistent with those reported during implantations of microdialysis probes [11] or intracranial electrodes [12]. Physiological changes such as gliosis are significant several days or weeks after implantation. In this study, microcoils are implanted for a very short period of time relative to the duration of the experiment (approximately 2–3 h). Note that, contrary to the microdialysis where gliosis on the membrane can be redhibitory in chronic implantation, the sensitive region of the coil extends at some distance from the microwires (few hundreds of micrometers) where the tissues are less likely to be impacted by the microcoil implantation.

The use of copper wire, with a magnetic volumic susceptibility (−9.63 ppm) close to human tissue (−9.2 to −8.8 ppm), allowed to limit the distortion of the magnetic field caused by the difference in susceptibility of the two media.

The gain in sensitivity obtained through the use of microcoils facilitates the detection of metabolic variations characteristic of tumor metabolism at submicroliter scale.

The mitotic activity of glioma cells can be assessed by the tCho/tCr ratio and necrosis by the increase in lipids and decrease in tCr. For C6 gliomas, Coquery et al. showed that the concentration of tCr in the tumor and in the healthy contralateral side remained relatively stable. The decrease of tCr in the spectrum of the C6 glioma-bearing rat seems to demonstrate that there was an onset of necrosis within the tumor. In general, in case of tumor proliferation, an increase in the tCho/NAA ratio is observed and is explained by the replacement of normal neurons by glial tumor cells. Particular metabolic and spectroscopic differences also occur in the differentiation of low and high-grade gliomas [13].

The gain in sensitivity obtained through the use of microcoils enables the detection of metabolic variations characteristic of tumor metabolism at submicroliter scale which can be a real asset in the context of tumors with a heterogeneous appearance. Indeed, tumors do not consist of a set of homogeneous cells but of a complex and very heterogeneous system [14]. The possibility of performing MRS or MRI in very small volumes of interest would make it possible to study different aspects of the tumor without having to perform a biopsy of the tumor, for example, which is more damaging to the integrity of the tissue. In the case of gliomas, there is a classical tumor organization in different layers—necrotic heart, inner crown of quiescent cells (which usually do not multiply) and proliferating cells on the outer surface. There is also an area, visible on MRI, that extends all around the tumor. This area commonly corresponds to an edema that also contains cancer cells but at a much lower density than in the tumor. The spectroscopic analysis, performed by these implantable microcoils, would allow to guide and evaluate therapeutic treatments to the most metabolically active areas, such as the tumor growth crown, with a high precision of the volume concerned, regardless of the anatomical aspect of the tumor.

In the case of metastases (often smaller than primary tumors) [15], the use of implantable NMR microcoils could also be advantageous. In fact, the spread of one or more metastases is very common in the progression of cancer. Approximately 20–40% of cancer patients are susceptible to develop a brain metastasis. According to several studies, the majority of deaths (at least 2/3) due to cancer are caused by metastasis [16]. A better understanding of the molecular processes involved in the formation of these metastases by using MRS and MRI would further help the development of therapeutic strategies such as immunotherapy [17], stereotactic radiosurgery [18] or combined techniques such as concomitant chemoradiotherapy [19] and thus reduce cancer mortality.

The main limitation of this study is the limited number of glioma-bearing animals investigated. This limited number of glioma-bearing rats does not allow to assess the reproducibility of metabolite quantification in tumor tissue. The reproducibility study of the use of implantable microcoils is, however, validated by the data obtained in the five healthy rats. In fact, the results obtained and presented in this article represent a feasibility and proof-of-concept study.

Following the positive results obtained and the validation of the approach and its applicability for the investigation of tumor metabolism on a very small scale, more advanced studies with modifications of protocols and instrumentation (active decoupling to improve the homogeneity of the RF excitation field, pre-amp integration) are being developed in the laboratory.

## 4. Materials and Methods

### 4.1. Microcoil Design and Manufacturing

**Figure 4:**
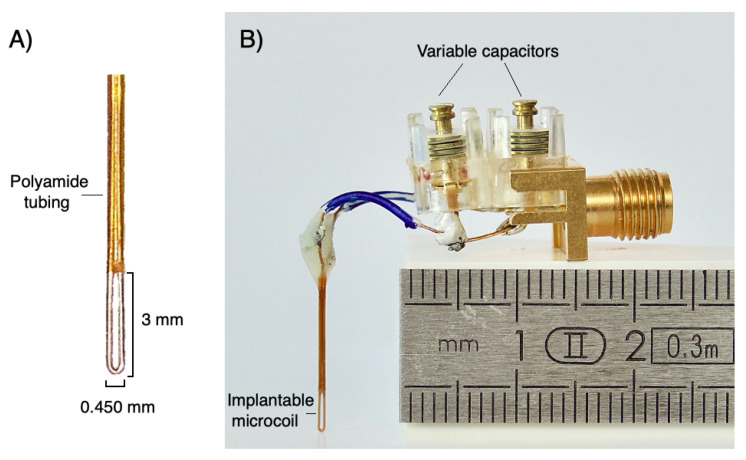
(**A**) Picture of an implantable microcoil and part of its polyamide tubing. (**B**) Overview of the main elements of the assembly including an implantable microcoil.

The microcoil described in this study was constructed with 150-μm-thick copper wire. The microcoil was an elliptical shaped loop with external dimensions of 3 mm in length, 0.450 mm in width and 0.150 mm in thickness, visible in Figure 4A.

The extremities of the copper wire were then wound one on top of the other and inserted into a 5 mm long biocompatible polyamide tubing with inner and outer diameters of 350 and 380 μm respectively. The endings of the microwire were then soldered on a printed circuit board approximately 1 cm away from the tubing ending. Constant and variable amagnetic capacitors were used to tune the microcoil at nucleus Larmor frequency of interest (300 MHz for ${}^{1}$H in a magnetic field of 7 T) and to match to the transmission impedance channel (50-ohm cable). They are soldered on the printed circuit board. The assembly is then connected to the preamplifier of the spectrometer via a coaxial cable. An overview of the assembly is shown in Figure 4B.

The detection volume of the implantable microcoil was estimated by simulations of the radiofrequency field distribution by the finite element method with the software COMSOL Multiphysics® (COMSOL Inc., Stockholm, Sweden) and MRI measurements using a ZTE sequence (FOV = 0.9 cm, isotropic resolution 70 μm/pixel). Results from RF simulations and ZTE acquisitions are presented in the Supplementary Materials (Supplementary Figures S1–S3).

### 4.2. Magnetic Resonance Experiments

MRI and MRS experiments were performed on a 7-T scanner (BioSpec 70/20, Bruker Biospin) with ParaVision (6.0.1) software. For radiofrequency excitation, we used a 86-mm inner diameter quadrature Bruker volume coil for in vivo experiments and a rat brain Bruker phased array surface coil for in vitro experiments (Bruker, Ettlingen, Germany). The implantable microcoils were centered and positioned vertically within the volume coil. The normal to the elliptical surface of the microcoil was placed perpendicular to the B${}_{0}$ static magnetic field. No active decoupling of the microcoil was used during RF excitation.

The magnetic field homogeneity was adjusted using the MAPSHIM method [20] acquired with the transmit volume coil followed by an iterative adjustment of the first and second order shims using the microcoil as a receiver.

Magnetic resonance spectra were acquired using a single-voxel PRESS (point-resolved spectroscopy) sequence with the following parameters—echo time TE = 15 ms; repetition time TR = 2000 ms; voxel size = 2 × 4 × 2 mm${}^{3}$. The water signal was suppressed by variable power RF pulses with optimized relaxation decays (VAPOR) [21]. MR spectra were postprocessed using the Totally Automatic Robust Quantitation in NMR (TARQUIN) method [22]. A first order phasing and a Lorentzian apodization of 5 Hz were performed and the residual water components were removed using the HLSVD (Hankel-Lanczos Singular Value Decomposition) algorithm [23].

To characterize the detection volume of the implantable microcoil, MRI acquisitions with the microcoil as a receiver only were performed using a ZTE (zero echo time) sequence with a repetition time of 2.5 ms and a total acquisition time of 2 min. Matrix size was 128 voxels, resulting in 70 μm isotropic resolution.

To control tumor growth and volume and to localize the position of the implanted microcoil in the brain during in vivo experiments, high resolution T${}_{2}$-weighted images were acquired using a Turbo RARE (rapid imaging with refocused echoes) sequence with the following parameters: echo time TE = 33 ms, repetition time TR = 2500 ms, flip angle 90°. Matrix size was 256 × 256, resulting in a pixel size of 0.14 × 0.14 mm${}^{2}$. 9 slices were acquired with a slice thickness of 0.8 mm (slice gap = 0 mm) using the volume coil as a transceiver.

### 4.3. In Vitro Experiments

The comparative study of performances of a conventional Bruker phased array surface coil and the microcoil was performed using a phantom containing a solution of aCSF (NaCl, 147 mM; KCl, 2.7 mM; CaCl${}_{2}$, 1.2 mM; MgSO${}_{4}$, 0.85 mM) and seven brain metabolites: choline (Cho), creatine (Cr), glutamate (Glu), lactate (Lac), myo-inositol (mI), N-acetylaspartate (NAA) and taurine (Tau) at the concentration of 25 mM. The microcoil was immersed in the phantom and the phased array surface coil was placed over the phantom whose dimensions were close to those of a rat brain. Both coils (Bruker phased array surface coil and microcoil) were used with the same experimental conditions (reception only, tuning and matching) and RF transmission was performed using the quadrature volume coil. NMR experiments were carried out at 7 T and for each coil, the same voxel, with dimensions 2 × 4 × 2 mm${}^{3}$, was selected by the PRESS sequence. For the surface coil acquisition, the voxel was positioned at a depth of 3 mm to simulate in vivo conditions and corresponded both to the excitation and the reception volume. For the microcoil acquisition, the voxel was centered around the microcoil and corresponded to the excitation volume only (the detection volume is given by the microcoil sensitive volume which is 450 nL).

Figures of merit are available to accurately determine the mass or concentration of metabolites required to obtain a desired SNR in a given acquisition time [7]. These performance parameters are time-normalized concentration sensitivity S${}_{c}$ and time normalized mass sensitivity S${}_{m}$:

(1) $$S_{c}=\frac{SNR}{C\cdot \sqrt{T_{acq}}}$$

(2) $$S_{m}=\frac{SNR}{mol\cdot \sqrt{T_{acq}}}$$

with mol, the number of moles observed in the detection volume of the coils, C, the concentration of molecules observed and, T${}_{acq}$, the acquisition time.

The time-normalized limits of detection in terms of concentration, nLOD${}_{c}$, and of mass, nLOD${}_{m}$, are therefore:

(3) $$nLOD_{c}=\frac{3\cdot C\cdot \sqrt{T_{acq}}}{SNR}=\frac{3}{S_{c}}$$

(4) $$nLOD_{m}=\frac{3\cdot mol\cdot \sqrt{T_{acq}}}{SNR}=\frac{3}{S_{m}}$$

Thus, the gain factor, FOG, of the microcoil is defined as follows:

(5) $$FOG_{microcoil}=\frac{nLOD_{m}\left(surface\;coil\right)}{nLOD_{m}\left(microcoil\right)}$$

These parameters represent a simple and reliable technique for indicating the performance of microcoils. The values of the S${}_{c}$, S${}_{m}$, nLOD${}_{c}$, nLOD${}_{m}$ and FOG indicators were measured for the choline, N-acetylaspartate, lactate doublet and creatine resonances. The SNR and spectrum FWHM (full width at half-maximum) values were obtained using the TopSpin software (Bruker, Ettlingen, Germany).

### 4.4. In Vivo Experiments

Male rats of Wistar strain (7 weeks of age, 160–180 g) were used for in vivo experiments. Animals were procured from Janvier Laboratory (Le Genest-Saint-Isle, France). They were kept in standard housing conditions (12 h light-dark cycles) with a standard rodent chow and water available *ad libitum*. All animal procedures were performed in accordance with the rules of the European Committee Council Directive 2010/63/ EU after validation by our local ethical committee and authorization from the French Ministry of Research (University of Bordeaux, reference number 04490.02).

C6 glioma-bearing rats were obtained by a stereotactic injection into the right barrel cortex with C6 glioma cells (10${}^{6}$ cells) derived from N-nitrosomethylurea-induced rat glioblastoma (purchased from the ATCC-LGC Bank, Manassas, VA, USA) under general anesthesia (2.5% isoflurane in a mixture of air/O${}_{2}$ (70/30)). Ten days later, the cannula hosting the implantable microcoil were implanted in the periphery of the tumor in the right barrel cortex for the C6 glioma-bearing rats and in the right barrel cortex for the sham rats. During surgery, rats were anesthetized via a facial mask and fixed in a stereotaxic frame. Following a midline incision, two small holes were drilled in the skull: one using a 1 mm diameter drill bit for the placement of the cannula (+3 mm medio/lateral right hemisphere according to the atlas of the rat brain and corresponding to the S1BF area) and one with a 1.3 mm drill bit for the plastic screw close to the previous hole. Screws and cannula were fixed on the skull with dental cement (Dentalon Plus, Kulzer, Germany) on the surface of the skull. The plastic screw increases the grip of the cement on the skull and thus consolidates the fixation of the cannula. After surgery, rats were housed individually and received doses of buprenorphine 0.05 mg/kg every 12 h for 48 h.

The NMR experiment took place one day after the surgery. The rat was anesthetized in an animal chamber using a gas mixture of O${}_{2}$ and isoflurane (3%). After lying the animal down in prone position in an animal bed, the detection loop and polyamide tubing assembly were inserted in the cannula. Only the detection loop was implanted in the brain of the animal. The animal bed was moved inside the magnet with the microcoil positioned at the magnetic isocenter. The animal was maintained under anesthesia using a gas mixture of O${}_{2}$ and isoflurane (1–2%). The body temperature was maintained around 37 °C by a warm water circuit. During the acquisition, physiological parameters were monitored to control the anesthesia and to record the state of the animal. A breathing sensor was placed under the animal and the breath rate was kept between 55 and 65 breaths per minute.

**Figure 5:**
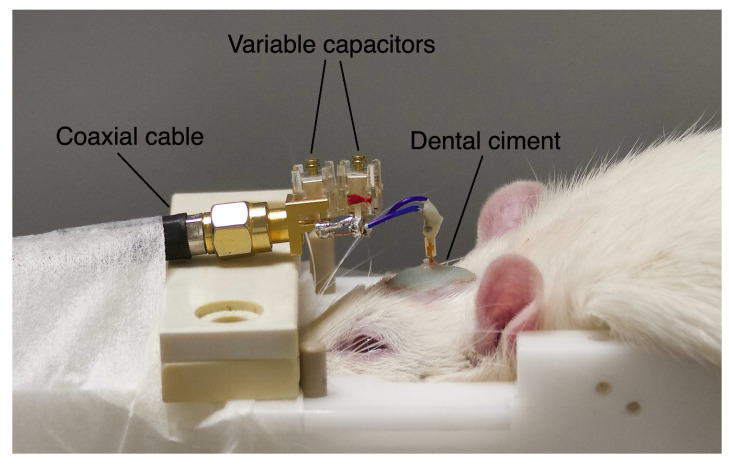
Illustration of the experimental set-up.

An overview of the anesthetized animal with the implanted microcoil is shown in Figure 5. A total of 10 animals (2 C6 glioma-bearing rats and 8 sham rats) were needed in this study to validate and optimize the surgery procedure, the spectroscopy and the imaging protocol using the implanted microcoils.

## 5. Conclusions

Small volume probes such as the implantable NMR microcoils presented in this study appear to be a very promising approach in the study of tumors.

This study has focused on the investigation of brain tumors but the use of these microcoils appears feasible for the study of other types of primary tumors and metastases in organs.

## Figures and Tables

**Table 1:** Sensitivities, S${}_{c}$ and S${}_{m}$, and limits of detection, nLOD${}_{c}$ and nLOD${}_{m}$, for the implantable microcoil and the surface coil measured for five resonance lines.

Probe	Detection Volume	Parameters	Lactate Doublet	NAA	Choline	Creatine	Creatine
			1.31 ppm	2.00 ppm	3.18 ppm	3.02 ppm	3.91 ppm
Implantable microcoil	0.450 µL	S${}_{c}$ SNR·mM${}^{-1}$min${}^{-1/2}$	0.12	0.34	0.80	0.29	0.23
		S${}_{m}$ SNR·µmol${}^{-1}$s${}^{-1/2}$	34.88	98.64	231.57	85.40	67.05
		nLOD${}_{c}$ mM·min${}^{1/2}$	24.67	8.72	3.71	10.07	12.83
		nLOD${}_{m}$ µmol·s${}^{1/2}$	0.08	0.03	0.01	0.03	0.04
Surface coil	16 µL	S${}_{c}$ SNR·mM${}^{-1}$min${}^{-1/2}$	0.07	0.14	0.30	0.12	0.12
		S${}_{m}$ SNR·µmol${}^{-1}$s${}^{-1/2}$	0.56	1.18	2.47	1.04	1.01
		nLOD${}_{c}$ mM·min${}^{1/2}$	42.62	20.30	9.78	23.18	23.73
		nLOD${}_{m}$ µmol·s${}^{1/2}$	5.28	2.52	1.21	2.87	2.94

**Table 2:** FOG${}_{microcoil}$ values averaged from five resonance lines.

Metabolite of Interest	Lactate Doublet	NAA	Choline	Creatine	Creatine
	1.31 ppm	2.00 ppm	3.18 ppm	3.02 ppm	3.91 ppm
FOG${}_{microcoil}$	61	83	93	81	65

## Data Availability

Data is contained within the article.

## Supplementary Materials

The following are available online at https://www.mdpi.com/2218-1989/11/3/176/s1, Figure S1: Characterization of the implantable microcoil using the finite element method (FEM) modeling software, Figure S2: Magnetic flux density norm (μT) along the red axis (present on the right figure) passing through the center of the microcoil (mm), Figure S3: ZTE acquisitions.
