# Supplementary material for: Implantable NMR Microcoils in Rats: A New Tool for Exploring Tumor Metabolism at Sub-Microliter Scale?
Source: Metabolites. 2021 Mar 17;11(3):176. doi: 10.3390/metabo11030176 (PMC8002894; doi:10.3390/metabo11030176)
Supplement: Supplementary file 1 [file metabolites-11-00176-s001.pdf]

metabolites

MDPI

Supplementary Materials

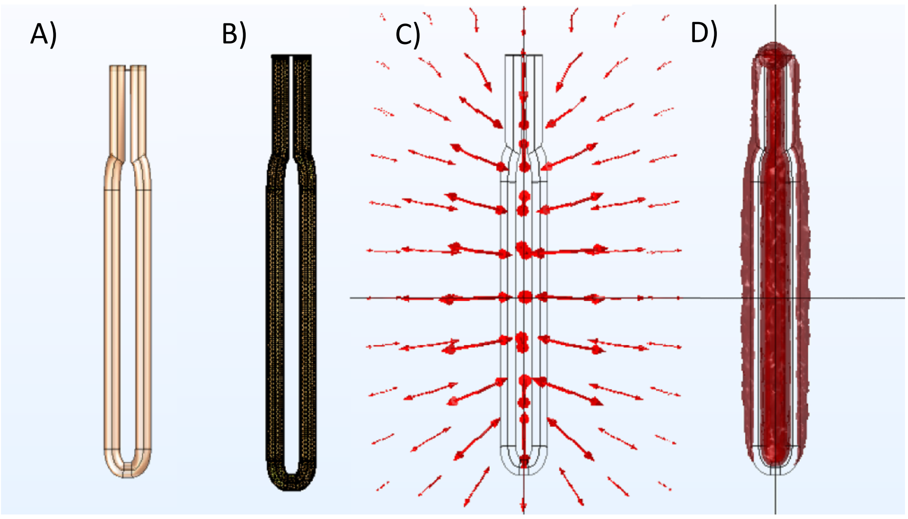

Supplementary Figure S1. Characterization of the implantable microcoil using the finite element method (FEM) modeling software.

(A) 3D view of the geometric construction of the microcoil. (B) 3D view of the microcoil mesh used for electromagnetic simulation. (C) 3D display of the B_1 magnetic field generated by the microcoil. Red arrows are proportional to the B_1 amplitude. (D) Isosurface of the amplitude of the transverse component of the B_1 RF field.

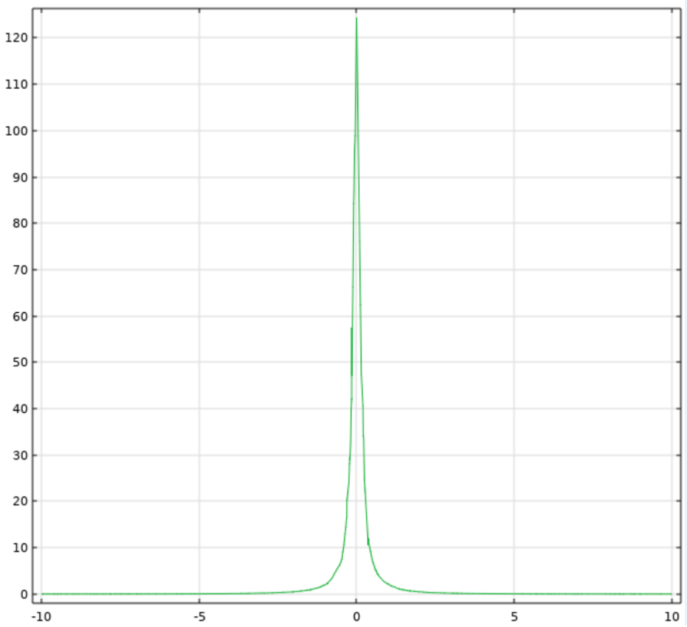

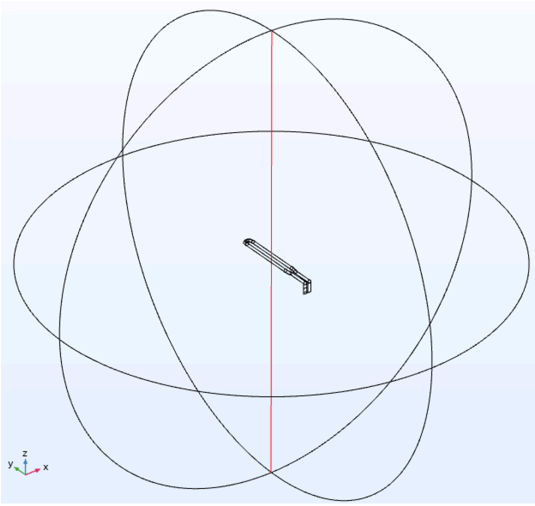

Supplementary Figure S2. Magnetic flux density norm (µT) along the red axis (present on the right figure) passing through the center of the microcoil (mm). This red axis is equal to the radius of the sphere (1cm) required for RF simulations with COMSOL Multiphysics.

The detection volume of the implantable microcoil was based on the spatial extension of the transverse B_1 RF field down to 33% of its maximum amplitude value. 33% of the maximum amplitude was empirically chosen as a cut-off for the limit of detection of the microcoil. The volume of the detection zone of the microcoil was approximated

to the volume of an ellipsoid (the volume of the copper microwires of the microcoil being substracted from this ellipsoid).

The volume of an ellipsoid is given by the formula: $V = \frac{4}{3}\pi\, a\, b\, c$ where a, b and c are the "semi-major axes". The values of a and b are the dimensions of the microcoil and the value of c is determined by the graph using the distance between 100 and 33% of the maximum Bz.

By calculation, the determined detection volume value was 0.459 µL which has been approximated at 450 nL.

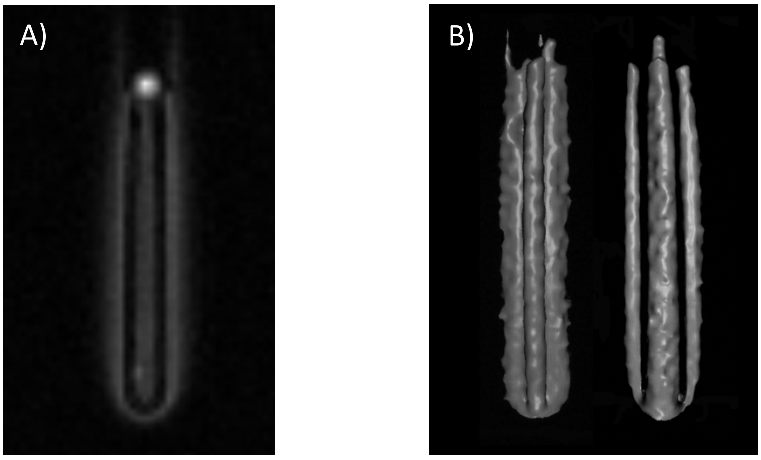

Supplementary Figure S3. ZTE acquisitions.

A) ZTE sagittal slice obtained with the implantable microcoil. B) Volume rendering of the 3D ZTE MRI acquisition using the implantable microcoil. The two views are rotated 90° with respect to each other around the vertical axis. Detection volume is calculated by summing the highlighted pixels in the 3D acquisition. The detection volume estimated by the MRI measurements was 476 nL which has been approximated at 450 nL.
